# P53 and p73 differ in their ability to inhibit glucocorticoid receptor (GR) transcriptional activity

**DOI:** 10.1186/1476-4598-5-68

**Published:** 2006-12-06

**Authors:** Lili Zhang, Linghu Nie, Carl G Maki

**Affiliations:** 1 Institute of Medicinal Biotechnology, Peking Union Medical College, Beijing, China; 2 Department of Radiation Oncology, University of Chicago, Chicago, IL 60637, USA

## Abstract

**Background:**

p53 is a tumor suppressor and potent inhibitor of cell growth. P73 is highly similar to p53 at both the amino acid sequence and structural levels. Given their similarities, it is important to determine whether p53 and p73 function in similar or distinct pathways. There is abundant evidence for negative cross-talk between glucocorticoid receptor (GR) and p53. Neither physical nor functional interactions between GR and p73 have been reported. In this study, we examined the ability of p53 and p73 to interact with and inhibit GR transcriptional activity.

**Results:**

We show that both p53 and p73 can bind GR, and that p53 and p73-mediated transcriptional activity is inhibited by GR co-expression. Wild-type p53 efficiently inhibited GR transcriptional activity in cells expressing both proteins. Surprisingly, however, p73 was either unable to efficiently inhibit GR, or increased GR activity slightly. To examine the basis for this difference, a series of p53:p73 chimeric proteins were generated in which corresponding regions of either protein have been swapped. Replacing N- and C-terminal sequences in p53 with the corresponding sequences from p73 prevented it from inhibiting GR. In contrast, replacing p73 N- and C-terminal sequences with the corresponding sequences from p53 allowed it to efficiently inhibit GR. Differences in GR inhibition were not related to differences in transcriptional activity of the p53:p73 chimeras or their ability to bind GR.

**Conclusion:**

Our results indicate that both N- and C-terminal regions of p53 and p73 contribute to their regulation of GR. The differential ability of p53 and p73 to inhibit GR is due, in part, to differences in their N-terminal and C-terminal sequences.

## Background

The p53 tumor suppressor pathway is inactivated in a majority of human cancers, either through mutation of the *p53 *gene or alterations of p53 regulators or p53-pathway proteins [1,2]. Wild-type p53 is a transcription factor that binds the promoter regions of various target genes in a sequence-specific manner and activates their transcription. Some of these target genes are necessary for p53 to induce cell cycle arrest following stress, such as *Waf1*, which encodes the cyclin-dependent kinase inhibitor protein p21 [3]. Other target genes are important for the apoptotic function of p53, including *Bax*, *Fas*/*Apo1*, *PUMA*, *Noxa*, and *Apaf1 *[4,5]. Cancer-derived mutations in p53 block its sequence-specific DNA-binding capability, resulting in decreased expression of these growth-inhibitory target genes. This can lead to uncontrolled cell growth and eventual carcinogenesis.

P73 is a p53-related protein that shares a high degree of amino acid sequence identity with p53 and many of the same structural features [6,7]. Given their similarities, it is important to determine whether p53 and p73 carry out similar or distinct functions, and whether they are regulated through similar or different mechanisms. Both p53 and p73 contain an N-terminal transactivation domain (TAD) and proline-rich domain (PRD), a central DNA-binding domain (DBD), and a C-terminal oligomerization domain (OD) [6,7]. P53 and p73 differ considerably in their extreme C-terminal sequences. The p53 extreme C-terminus (residues 364–393) is a basic-charged region that contains a cluster of six lysine residues. These lysines are sites of post-translational modifications (acetylation, ubiquitination, neddylation, methylation) that can regulate p53 stability and transcriptional activity [8-12]. In contrast, p73 contains an extended C-terminus that can vary in length due to alternative splicing, and that has no sequence or structural homology with p53. P53 and p73 can carry out some redundant functions. For example, p73 can bind to and activate various p53 target genes, and can induce growth arrest or apoptosis when over-expressed [13,14]. Further, levels of endogenous p73 protein increase in response to certain stresses, as does p53, and this p73 can induce apoptosis in p53-null cells [15,16]. Despite these similarities, however, the consequence of p53 or p73 loss on development and cancer susceptibility is strikingly different. P53 loss-of-function mutations are found in over 50% or all human cancers, and p53-deficient mice develop multiple cancers and die at an early age [17-19]. This is consistent with p53s role as a *bona fide *tumor suppressor. In contrast, mutations in p73 are not commonly associated with cancer, and p73-deficient mice display neurological, pheromonal, and inflammatory defects without an apparent increased cancer incidence [20]. These findings and others have suggested that p73 could play distinct roles in development that are not attributed to p53.

Glucocorticoid receptor (GR) is a nuclear receptor and ligand-dependent transcription factor (reviewed in [21,22]). GR belongs to the superfamily of steroid nuclear receptors that also includes estrogen receptor (ER), androgen receptor (AR), and progesterone receptor (PR). In the absence of ligand, GR is inactive and resides in the cytoplasm in complex with chaperones such as Hsp90. Ligand-binding promotes dissociation of GR from cytoplasmic complexes and its translocation to the nucleus, where it can then activate transcription of its target genes. The effect of activating GR appears to be cell-type specific. GR activation has been reported to promote survival and inhibit apoptosis in mammary epithelial cells, breast cancer cells, neuroblastoma cells, and other cell types [23-28]. In contrast, GR activation in thymocytes triggers apoptosis (reviewed in [29]). There is compelling evidence for cross-talk between GR and p53. Binding between GR and p53 has been demonstrated both *in vitro *and *in vivo*, and their interaction can lead to the mutual inhibition of both proteins [27,28]. The GR ligand dexamethasone has been reported to enhance binding between GR and p53 and, under certain conditions, to cause their cytoplasmic sequestration and degradation [28]. Complexes of p53 and GR may also contain MDM2, a p53-responsive protein that can act as an E3 ubiquitin ligase to promote degradation of both p53 and GR [28]. These findings have suggested that GR could enhance survival by sequestering p53 in the cytoplasm and, conversely, that p53 might enhance cell death by sequestering GR in the cytoplasm and blocking its survival function. To date, neither physical nor functional interactions between GR and p73 have been described.

In the current study, we examined whether p73 could bind GR and participate in negative cross-talk with GR, similar to p53. We found that both p53 and p73 could bind GR, and that p53 and p73-mediated transcriptional activity was inhibited by GR co-expression. Wild-type p53 efficiently inhibited GR transcriptional activity in cells expressing both proteins. Surprisingly, however, p73 was either unable to effectively inhibit GR, or increased GR activity slightly. To examine the basis for this difference, we generated a series of p53:p73 chimeric proteins in which corresponding regions of either protein have been swapped. These studies revealed that N- and C-terminal regions of p53 and p73 contribute to their regulation of GR. Fusion of p53 N- and C-terminal sequences to p73 allowed p73 to inhibit GR and, conversely, fusion of p73 N- and C-terminal sequences to p53 prevented p53 from inhibiting GR. We conclude that the differential ability of p53 and p73 to inhibit GR is due, in part, to differences in their N-terminal and C-terminal sequences.

## Results

### Both p53 and p73 can bind GR

**Figure 1:**
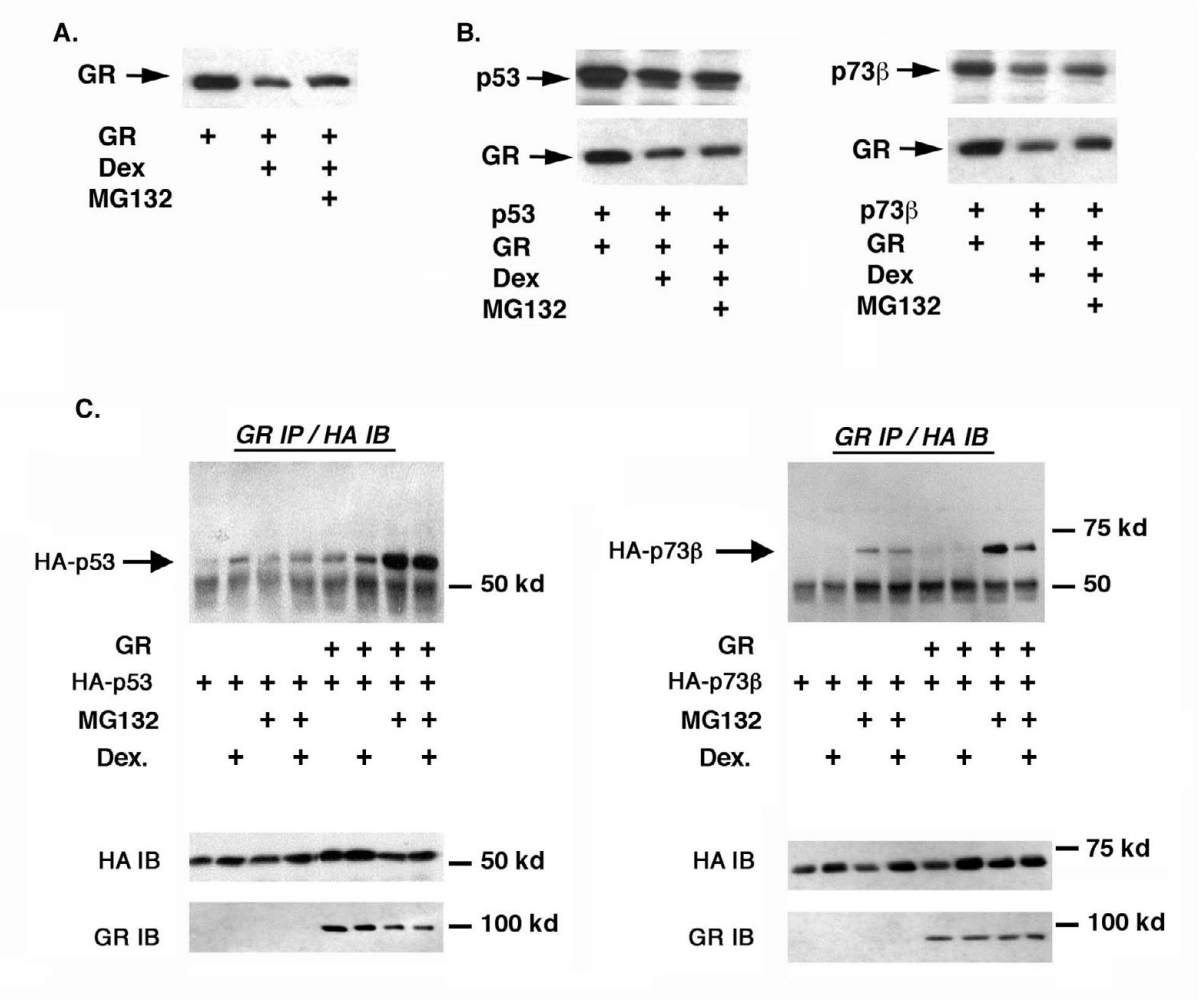
**Glucocorticoid receptor (GR) binding to p53 and p73**. *A *and *B*, Saos-2 cells (p53-null) were transfected overnight with 200 ng GR DNA and 100 ng DNA encoding either p53 or p73β. Where indicated, transfected cells were treated with dexamethasone (Dex, 100 nM) for 17 hrs and then incubated in the presence or absence of MG132 (30 μM) for an additional 6 hrs. GR, p53, and p73 levels were monitored by immunblotting. *C*, Saos-2 cells were transfected with DNAs encoding GR, HA-p53, or HA-p73β (1 μg each) as indicated. Transfected cells were either untreated (no tr), treated with dexamethasone (+Dex, 100 nM) for 24 hrs, treated with MG132 (30 μM) for 6 hrs, or treated with dexamethasone for 17 hrs followed by incubation in dexamethasone plus MG132 for an additional 6 hrs. Cell lysates were immunoprecipitated with GR polyclonal antibody, followed by immunoblotting with a HA monoclonal antibody. The position of HA-p53 (*left*) and HA-p73β (*right*) that co-immunoprecipitated with GR is indicated. The asterisk indicates detection of the antibody heavy chain used in the immunoprecipitation. Levels of GR, HA-p53, and HA-p73 in transfected cell lysates determined by immunoblotting without prior immunoprecipitation are shown in the lower panels.

P53, p73, and GR are each short-lived proteins that undergo proteasome-dependent degradation [30-32]. GR is targeted for proteasomal degradation in the presence of its activating ligand dexamethassone (Dex) [28]. P53 can bind GR and has also been reported to undergo enhanced degradation upon Dex-treatment [28]. We wished to test whether p73 can also bind GR. First, Saos-2 cells (p53-null) were transfected with DNAs encoding either GR only (Fig. 1A), or co-transfected with GR and p53 or p73β (Fig. 1B). Cells were then either untreated or treated with Dex for 17 hrs, followed by incubation for an additional 6 hrs in the presence or absence of the proteasome inhibitor MG132. GR levels decreased with Dex treatment when expressed alone or with p53 or p73β, and this decrease was partially blocked by MG132. P53 and p73 levels were similarly decreased in Dex treated cells, and this decrease was also partially blocked by MG132-treatment (Figs 1A and 1B). The results suggest Dex treatment may enhance proteasomal degradation of p53, p73, and GR under these conditions.

We next asked whether binding between GR and either p53 or p73 could be detected. For this, Saos-2 cells were transfected with expression DNAs (1 μg each) encoding GR and HA-tagged p53 or p73β. Transfected cells were then either untreated or treated with Dex for 17 hrs, followed by incubation for an additional 6 hrs in the presence or absence of MG132 to block their potential degradation. Cell lysates were immunoprecipitated with a GR antibody, followed by immuno-blotting with an HA antibody to detect co-immunoprecipitating p53 or p73β. As shown in Fig. 1C, both p53 and p73β co-immunoprecipitated with GR. Interestingly, binding between transfected GR and p53 or p73β was most evident in cells treated with MG132, suggesting the ability to detect GR complexes with p53 or p73 may be limited by their proteasomal degradation. p53 appeared to bind GR to a slightly greater extent than did p73β.

### GR inhibits p53 and p73 transcriptional activity

**Figure 2:**
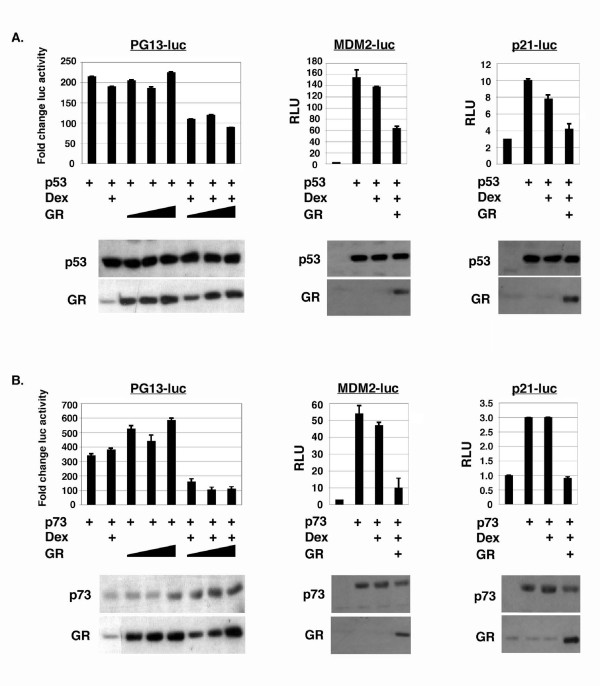
**GR inhibits p53 and p73 transcriptional activity**. *A*, Saos-2 cells were transfected with DNAs encoding p53 (50 ng), the indicated p53/p73-responsive reporter DNA (pG13-luc, p21-luc, or MDM2-luc, 100 ng each), and GR (100, 250, 500 ng for pG13-luc; 500 ng for p21-luc and MDM2-luc). 24 hrs after transfection, cells were untreated or treated with dexamethasone (100 nM) for an additional 20 hrs. Luciferase activity in transfected cell lysates was determined. The fold change in pG13-luc activity is plotted (+/- standard error of the mean, s.e.m.) from multiple experiments compared to the activity of pG13-luc alone, whose value is considered 1.0. Relative luciferase activity (RLU) is plotted (+/- s.e.m.) for MDM2-luc and p21 luc. Representative immunoblots for each experiment are shown. *B*, Same as in *A *above, but with transfection of p73β DNA (50 ng).

Given that GR can bind p53 and p73, we wished to test whether GR could inhibit p53 or p73 transcriptional activity. To this end, Saos-2 cells were transfected with different p53 and p73-responsive luciferase reporter genes (pG13-luc, MDM2-luc, and p21-luc) either alone, or with p53, p73 and GR in different combinations. p53 or p73-dependent transactivation of these reporter genes was assessed. As shown in Figs. 2A and 2B, p53 and p73 activated expression of each reporter gene. Co-expression of GR without Dex treatment, or treatment with Dex alone, had little or no effect on this p53 or p73-dependent activation. However, p53 and p73 activity was inhibited in each case when cells co-expressing GR were also treated with Dex. This indicates that GR, in the presence of its ligand (Dex), can inhibit the transcriptional activity of both p53 and p73.

### P53, but not p73, can efficiently inhibit GR transcriptional activity

**Figure 3:**
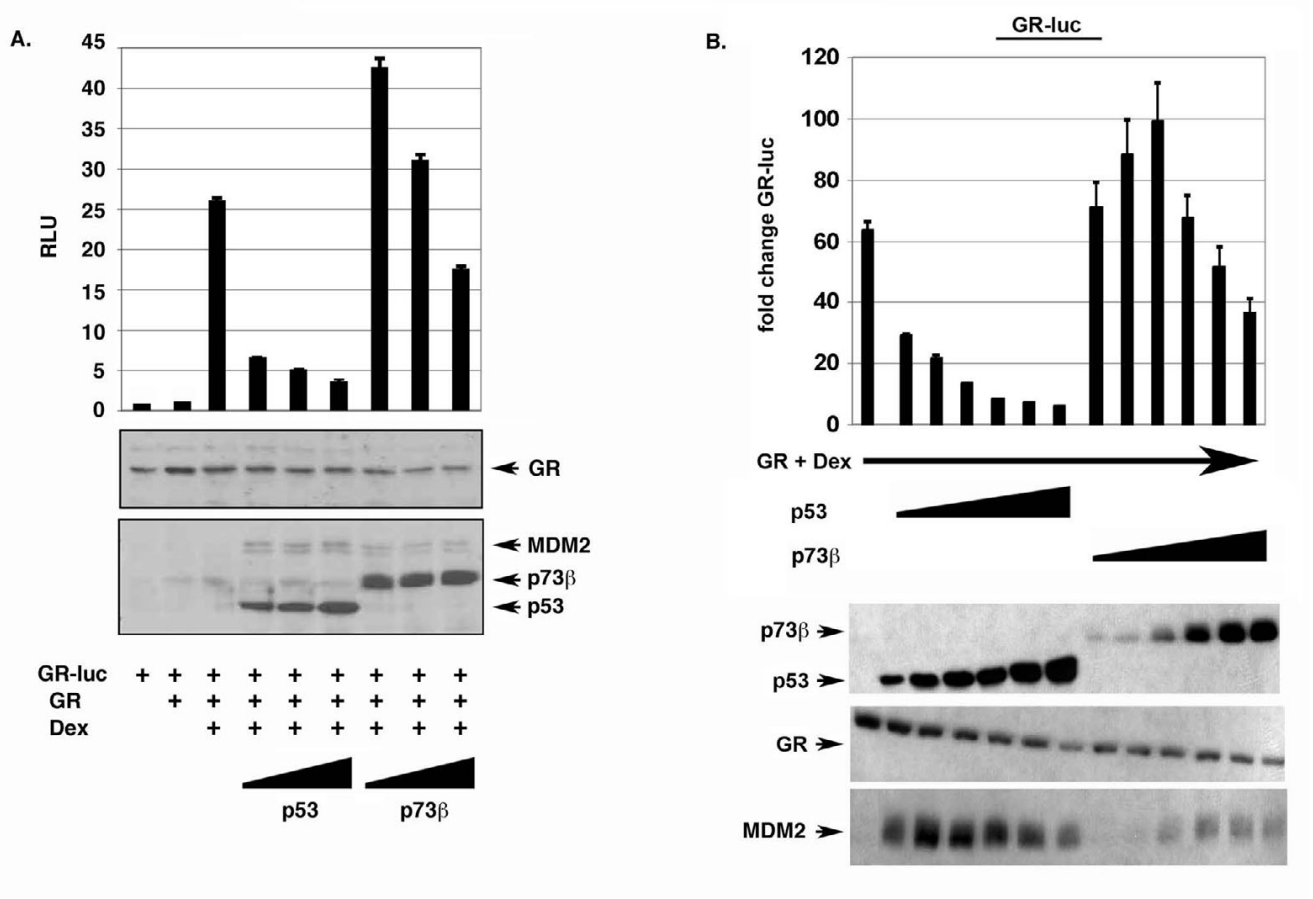
**p53 and p73 differ in their ability to inhibit GR transcriptional activity**. *A*, Saos-2 cells were transfected with the GR-responsive luciferase reporter GR-luc (200 ng), and DNA encoding GR (100 ng), and increasing amounts of HA p53 or HA p73β DNA (250, 500, 1000 ng) as indicated. 24 hrs after transfection, cells were untreated or treated with dexamethasone (100 nM) for an additional 20 hrs. Luciferase activity in transfected cell lysates was determined and relative luciferase activity (RLU) is plotted from triplicate experiments (+/- s.e.m.). *Lower *Representative immunoblot shows p53, p73, GR, and MDM2 expression levels in this experiment. *B*, Saos-2 cells were transfected with the GR responsive reporter GR-luc (200 ng) and DNA encoding GR (100 ng), and increasing amounts of Flag p53 or Flag p73β DNA (50, 100, 250, 500, 750, 1000 ng) as indicated. 24 hrs after transfection, cells were untreated or treated with dexamethasone (100 nM) for an additional 20 hrs. Luciferase activity in transfected cell lysates was determined and are plotted. The fold change in GR-luc activity is plotted (+/- s.e.m.) from multiple experiments compared to the activity of GR-luc alone, whose value is considered 1.0. *Lower*, Representative immunoblot shows GR, p53, p73, and MDM2 protein levels in transfected cell lysates.

P53 has been reported to inhibit the transcriptional activity of GR [28,33]. To test whether p73 can also inhibit GR activity, we monitored activation of a GR-responsive luciferase reporter gene (GR-luc, Fig. 3) when GR was co-expressed with p53 or p73. GR-luc was not activated by GR expression alone (Fig. 3A) or by Dex treatment alone. However, as shown in Fig. 3A, GR-luc was highly activated when cells transfected with GR expression DNA were treated with Dex. Expression of p53 at increasing amounts inhibited the transcriptional activity of GR (Figs. 3A and 3B), consistent with previous results. In contrast, and surprisingly, co-expression with increasing amounts of p73 inhibited GR either less efficiently than p53 or not at all, and low levels of transfected p73 actually increased GR activity (Figs. 3A and 3B). Thus, p53 and p73 differ in their abilities to inhibit GR transcriptional activity.

### P73 fails to inhibit GR when MDM2 is over-expressed

**Figure 4:**
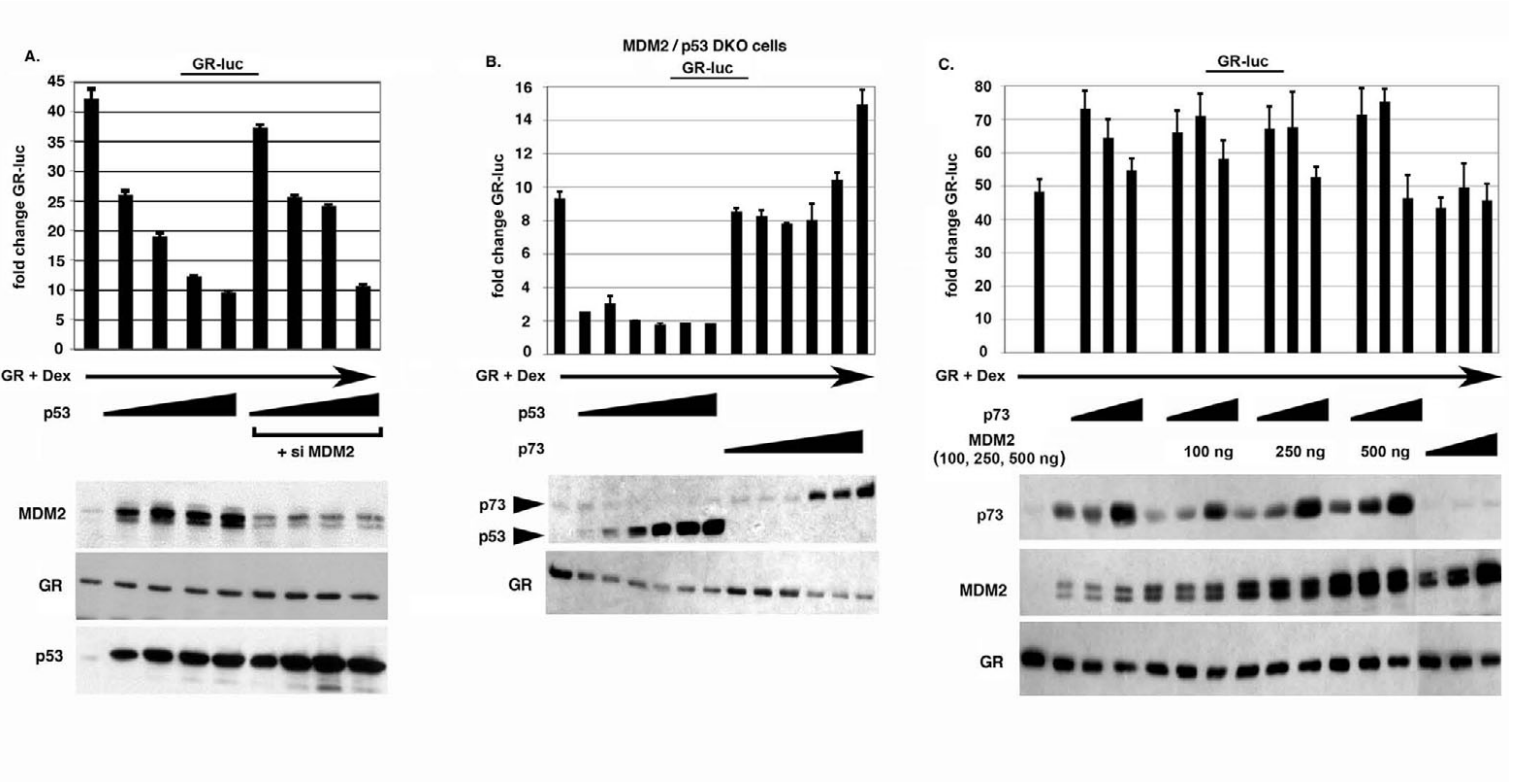
**Role of MDM2 in GR inhibition by p53 and p73**. *A*, Saos-2 cells were transfected with GR-luc DNA (200 ng) and increasing amounts of DNA encoding p53 (100, 250, 500, 750 ng). Where indicated cells were also transfected with DNA encoding siRNA directed against MDM2 (si MDM2). 24 hrs after transfection, cells were treated with dexamethasone (100 nM) for an additional 20 hrs, and luciferase activity in transfected cell lysates determined. Fold change in GR-luc activity is plotted (+/- s.e.m.) from multiple experiments compared to the activity of GR-luc alone, whose value is considered 1.0. GR, MDM2, and p53 immunoblots are shown below. *B*, MDM2/p53 double knockout (DKO) cells were transfected with GR-luc DNA (200 ng) and increasing amounts of DNA encoding p53 or p73 (50,100, 250, 500, 750, 1000 ng). Cells were treated with dexamethasone (100 nM) as described above, and luciferase activity in transfected cell lysates determined. Fold change in GR-luc activity is plotted compared to the activity of GR-luc alone, whose value is considered 1.0. Representative GR, p53 and p73 immunoblots are shown below. *C*, Saos-2 cells were transfected with GR-luc DNA (200 ng) and increasing amounts DNA encoding p73 (250, 500, 1000 ng). Where indicated cells were also transfected with the indicated amount DNA encoding MDM2 (250, 500, 1000 ng). Cells were treated with dexamethasone (100 nM) as described above and luciferase activity determined. Fold change in GR-luc activity is plotted. Representative GR, MDM2, and p73 immunoblots are shown below.

Wasylyk and colleagues reported that p53 may inhibit GR through activation of MDM2 [28]. According to this model, p53 activates MDM2 gene expression, and increased levels of MDM2 protein then promotes GR and p53 degradation. We observed lower induction of MDM2 in cells expressing p73 compared to cells expressing p53 (Figs. 3A and 3B), suggesting that the relative inability of p73 to inhibit GR might be due to its less efficient induction of MDM2. To test this possibility, we first monitored p53 inhibition of GR under conditions where endogenous MDM2 expression was inhibited by siRNA (Fig. 4A). The results show that p53 could still inhibit GR under conditions where MDM2 levels were decreased by siRNA, though knockdown of MDM2 diminished p53 inhibition of GR slightly. This suggested MDM2 may contribute to GR inhibition by p53, but is not absolutely required. Next, we monitored GR inhibition by p53 and p73 in MDM2/p53 double knockout (DKO) cells. p53 efficiently inhibited GR activity in these p53/MDM2 DKO cells, whereas p73 did not (Fig. 4B). Thus, the different ability of p53 and p73 to inhibit GR is observed even in the absence of MDM2 expression. Finally, we asked whether artificially increasing MDM2 levels would allow p73 to inhibit GR activity. To this end, GR activity was monitored in cells expressing increasing amounts of p73 either alone, or with increasing amounts of MDM2 (Fig. 4C). p73 was unable to inhibit GR activity when expressed alone or with MDM2, and MDM2 alone also had no effect on GR activity. Based on these results it appears that the differential ability of p53 and p73 to inhibit GR activity does not result from lesser induction of MDM2 by p73.

### Chimeric p53:p73 proteins have altered abilities to inhibit GR

**Figure 5:**
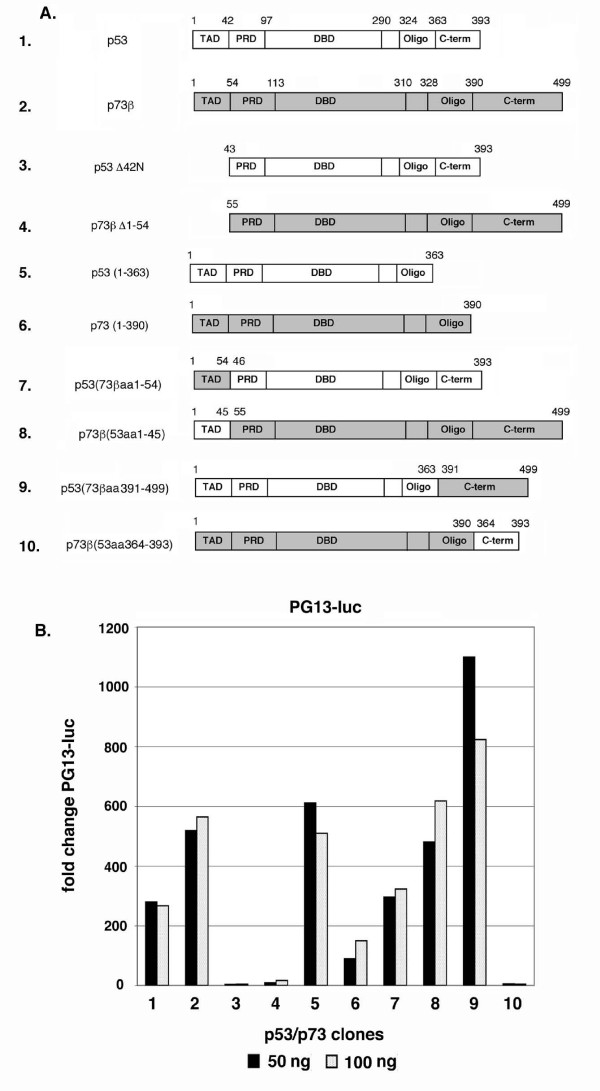
**p53 and p73 chimeric proteins**. *A*, Schematic of the Flag-tagged p53 and p73 wild-type (wt) and chimeric proteins. TAD = N-terminal transactivation domain, PRD = proline-rich domain, DBD = DNA-binding domain,, oligo = oligomerization domain, C-term = C-terminal domain. In this diagram, clear (unfilled) areas are from p53, and gray (filled) areas are from p73β. Numbers indicate the amino acid sequence positions in each protein. *B*, Saos-2 cells were co-transfected with 100 ng pG13-luc DNA and 50 or 100 ng DNA encoding the indicated protein from *A*. The fold change in pG13-luc activity is plotted compared to the activity of pG13-luc alone, whose value is considered 1.0. Results are the average from two experiments.

P53 and p73 share considerable amino acid sequence and structural similarity. We considered that one or more unique sequence elements in p53 or p73 might account for their different abilities to inhibit GR. To explore this possibility, a series of p53:p73 chimeric proteins were generated in which corresponding regions of either protein have been swapped (Figs. 5A and 8A). The high degree of structural homology between p53 and p73 should allow one to switch the various domains of each protein without disrupting protein conformation. A similar approach was used by Yuan et al. to identify specific sequence elements in p53 that confer degradation by MDM2 [34]. Deletion mutants of p53 and p73 were also generated that lack the conserved N-terminus of each protein (p53 Δ1–42 and p73 Δ1–54) or the unique C-terminus of each protein [p53 (1–363) and p73 (1–390)] (Fig. 5A). Transcriptional activity of each clone was tested by monitoring activation of the p53 reporter gene pG13-luc in transfected cells (Figs. 5B and 8B). p53 (1–363) had increased transcriptional activity compared to wild-type p53 (compare clones 1 and 5, Fig. 5B), consistent with early and more recent reports that the p53 C-terminus harbors a transcription inhibitory domain [35,36]. In contrast, p73 (1–390) had lower transcriptional activity compared to wild-type p73β, consistent with reports that the p73 C-terminus harbors a transcriptional activation domain (TAD) [37]. Similarly, p53 with the p73 C-terminus (clone 9, Figs. 5A and 5B) had high transcriptional activity, consistent with the transcription inhibitory domain of p53 being replaced with the C-terminal transcriptional activation of p73. Finally, p73 with the p53 C-terminus (clone 10, Figs. 5A and 5B) had very low activity, consistent with replacement of its C-terminal transcriptional activation domain with the transcription inhibitory domain of p53. As expected, p53 and p73 that lacked N-terminal sequences (p53 Δ1–42 and p73 Δ1–54) lacked transcriptional activity due to deletion of their N-terminal TAD.

**Figure 6:**
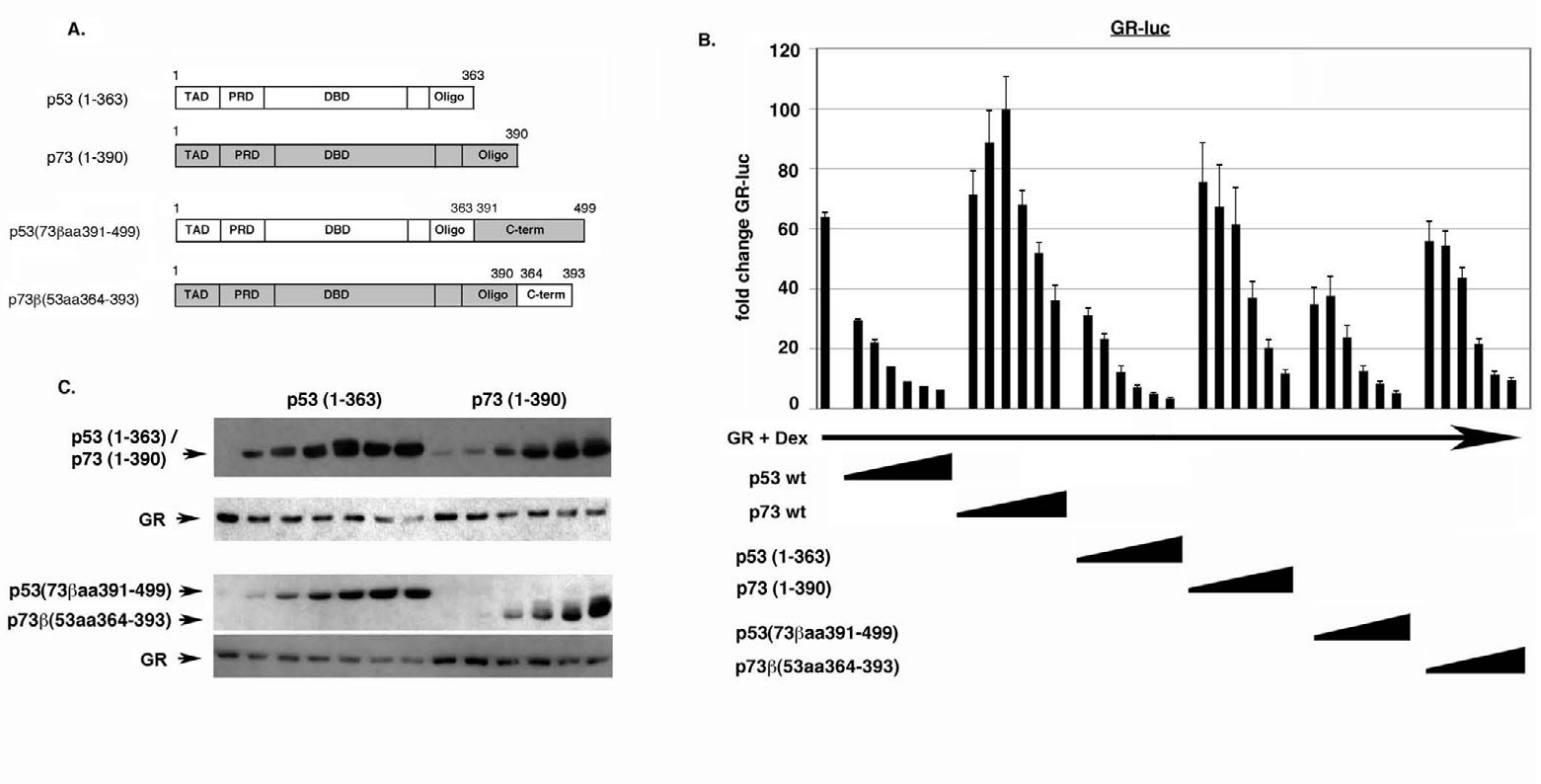
**C-terminal sequences in p53 and p73 affect their ability to inhibit GR**. *A*, Schematic of p53 and p73 C-terminal deletion mutants (ΔC) and C-term swap mutants. *B*, Saos-2 cells were transfected with the GR-responsive luciferase reporter GR-luc (200 ng), GR DNA (100 ng), and increasing amounts DNA encoding the indicated p53:p73 protein (50 – 1000 ng of each DNA). Transfected cells were treated with Dex (100 nM) as described earlier, and luciferase activity in transfected cell lysates was determined. Fold change in GR-luc activity is plotted (+/- s.e.m.) from multiple experiments compared to the activity of GR-luc alone, whose value is considered 1.0. *C*, Representative immunoblots show the level of each protein.

To examine the role that p53/p73 C-terminal sequences may play in GR inhibition, we monitored GR activity when co-expressed with the C-terminal deletion mutants of p53 and p73. As shown in Fig. 6B, p53 (1–363) inhibited GR equally well as p53 wt. Interestingly, p73 (1–390) did not increase GR activity as p73 wt did, and gained some ability to inhibit GR compared to p73 wt, especially at high input amounts. When equal protein expression levels were compared (as determined by densitometric scanning of films), p73 (1–390) had a slightly lesser ability to inhibit GR than p53 (1–363). These results indicate that 1) p53 C-terminal sequences are not absolutely required to inhibit GR, and 2) that p73 C-terminal sequences may contribute to GR activation by p73, and may limit p73s ability to inhibit GR. Next, we examined GR inhibition by the p53:p73 chimeric proteins in which the C-terminal sequences (C-term) have been swapped (Fig. 6B). p53 with the p73 C-term [p53(73βaa391–499)] was still able to inhibit GR activity (Fig. 6B). In contrast, p73 with the p53 C-term [p73β(53aa363–393)] had a slightly greater inhibitory effect on GR when compared with p73 (1–390). Based on these results it appears that differences in the unique C-terminal sequences of p53 and p73 may only partially explain their different abilities to inhibit GR. It is important to note there is not a strict relationship between the transcriptional activity of p53 or p73 and their ability to inhibit GR. For example, p53 (1–363) had increased transcriptional activity compared to p53 wt, but did not display increased ability to inhibit GR, while p73 (1–390) had decreased transcriptional activity compared to p73 wt but inhibited GR to a slightly greater extent. Similarly, p53 with the p73 C-terminus had the highest transcriptional activity but inhibited GR equally or slightly less well than p53 wt, while p73 with the p53 C-terminus had very little activity but inhibited GR to a greater extent than p73 wt.

**Figure 7:**
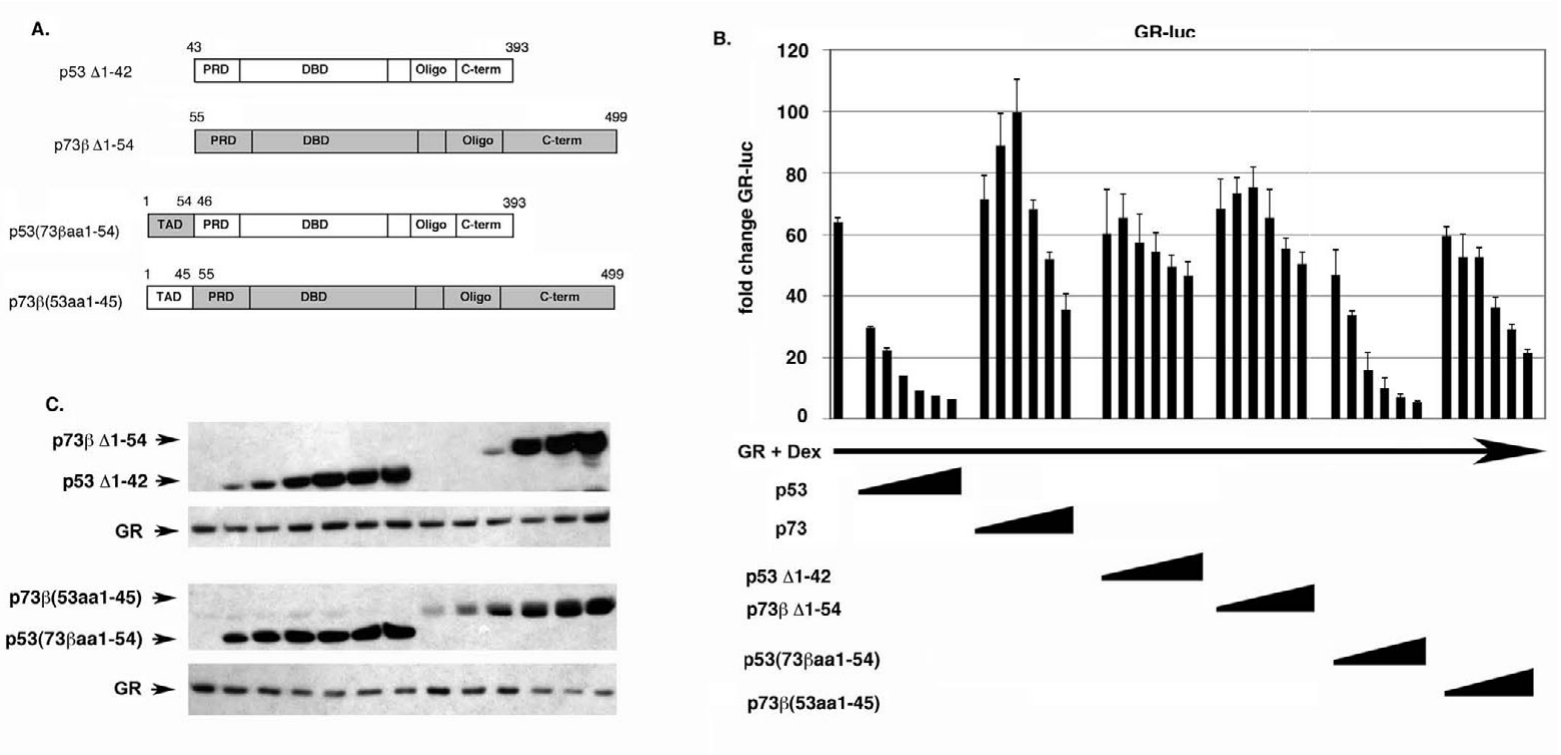
**N-terminal sequences in p53 and p73 affect their ability to inhibit GR**. *A*, Schematic of p53 and p73 N-terminal deletion mutants (ΔN) and N-terminal TAD swap mutants. *B*, Saos-2 cells were transfected with the GR-responsive luciferase reporter GR-luc (200 ng), GR DNA (100 ng), and increasing amounts DNA encoding the indicated p53:p73 protein (50 – 1000 ng of each DNA). Transfected cells were treated with Dex (100 nM) as described earlier, and luciferase activity in transfected cell lysates was determined. Fold change in GR-luc activity is plotted (+/- s.e.m.) from multiple experiments compared to the activity of GR-luc alone, whose value is considered 1.0. *C*, Representative immunoblots show the level of each protein.

To examine the role that p53/p73 N-terminal sequences may play in GR inhibition, we first monitored GR activity when co-expressed with N-terminal deletion mutants of p53 or p73. As shown in Fig. 7B, p53 Δ1–42 was unable to inhibit GR at all input amounts. p73 Δ1–54 was also unable to inhibit GR and, interestingly, also did not increase GR activity at low input amounts as p73 wt did. These results indicate that 1) p53 N-terminal sequences included within the N-terminal TAD are required for p53 to inhibit GR, and 2) that p73 N-terminal sequences may contribute to the slight activation of GR that is observed with p73 wt expression. Next, we examined GR inhibition by the p53:p73 chimeric proteins in which their N-terminal TAD sequences have been swapped (Fig. 7B). p53 with the p73 TAD [p53(73βaa1–54)] was still able to inhibit GR activity, though somewhat less well than p53 wt at low input amounts (Fig. 7B). In contrast, p73 with the p53 TAD [p73β(53aa1–45)] gained some ability to inhibit GR, especially at high input amounts. When equal protein expression levels were compared (as determined by densitometric scanning), p73β(53aa1–45) was slightly less able to inhibit GR than p53(73βaa1–54). Based on this it seems the different abilities of p53 and p73 to inhibit GR may also be explained partially, but not fully, by differences in their N-terminal TAD sequences.

**Figure 8:**
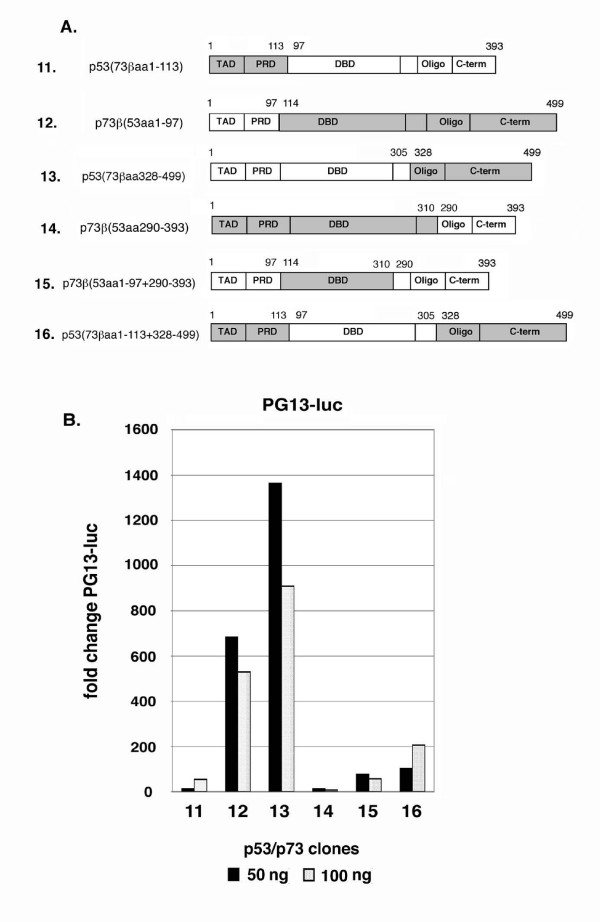
**p53 and p73 chimeric proteins with larger N- and C-terminal swaps**. *A*, Schematic of the p53 and p73 chimeric proteins in which larger portions of the N- and C-terminus have been swapped. *B*, Saos-2 cells were co-transfected with 100 ng pG13-luc DNA and 50 or 100 ng DNA encoding the indicated protein from *A*. The fold change in pG13-luc activity is plotted compared to the activity of pG13-luc alone, whose value is considered 1.0. Results are the average from two experiments.

**Figure 9:**
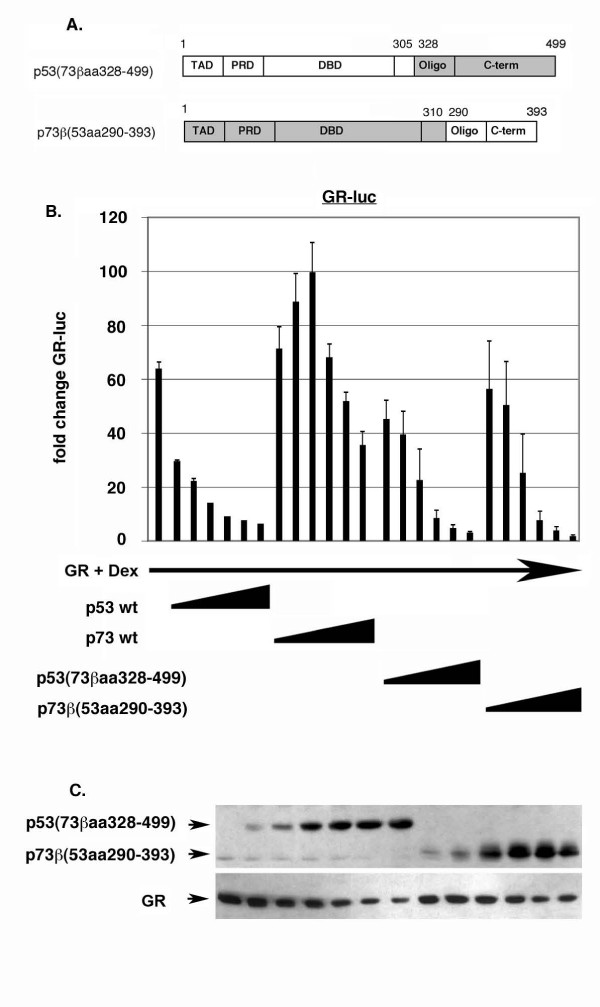
**C-terminal sequences affect p53 and p73 inhibition of GR**. *A*, Schematic of p53 and p73 C-terminal swap mutants in which the oligo and C-term have been swapped. *B*, Saos-2 cells were transfected with the GR-responsive luciferase reporter GR-luc (200 ng), GR DNA (100 ng), and increasing amounts DNA encoding wt p53 or p73, or the indicated p53:p73 C-terminal swap mutants (50 – 1000 ng of each DNA). Transfected cells were treated with Dex (100 nM) as described earlier, and luciferase activity in transfected cell lysates determined. Fold change in GR-luc activity is plotted (+/- s.e.m.) from multiple experiments compared to the activity of GR-luc alone, whose value is considered 1.0. *C*, Representative immunoblots show the level of each protein.

**Figure 10:**
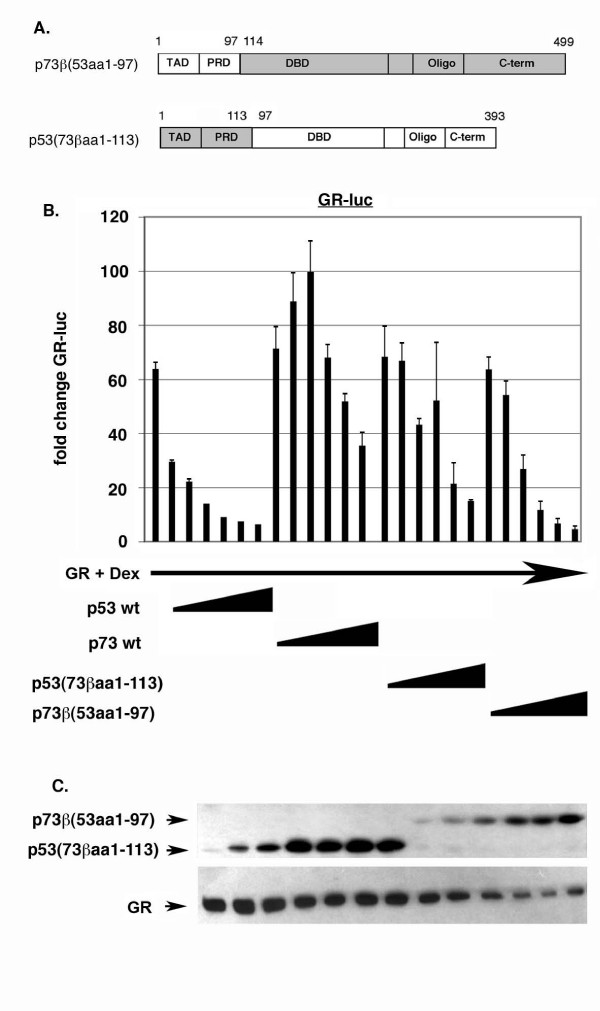
**N-terminal sequences affect p53 and p73 inhibition of GR**. *A*, Schematic of p53 and p73 N-terminal swap mutants in which the N-terminal TAD and PRD have been swapped. *B*, Saos-2 cells were transfected with the GR-responsive luciferase reporter GR-luc (200 ng), GR DNA (100 ng), and increasing amounts DNA encoding the indicated p53:p73 protein (50 – 1000 ng of each DNA). Transfected cells were treated with Dex (100 nM) as described earlier, and luciferase activity in transfected cell lysates was determined. Fold change in GR-luc activity is plotted (+/- s.e.m.) from multiple experiments compared to the activity of GR-luc alone, whose value is considered 1.0. *C*, Representative immunoblots show the level of each protein.

**Figure 11:**
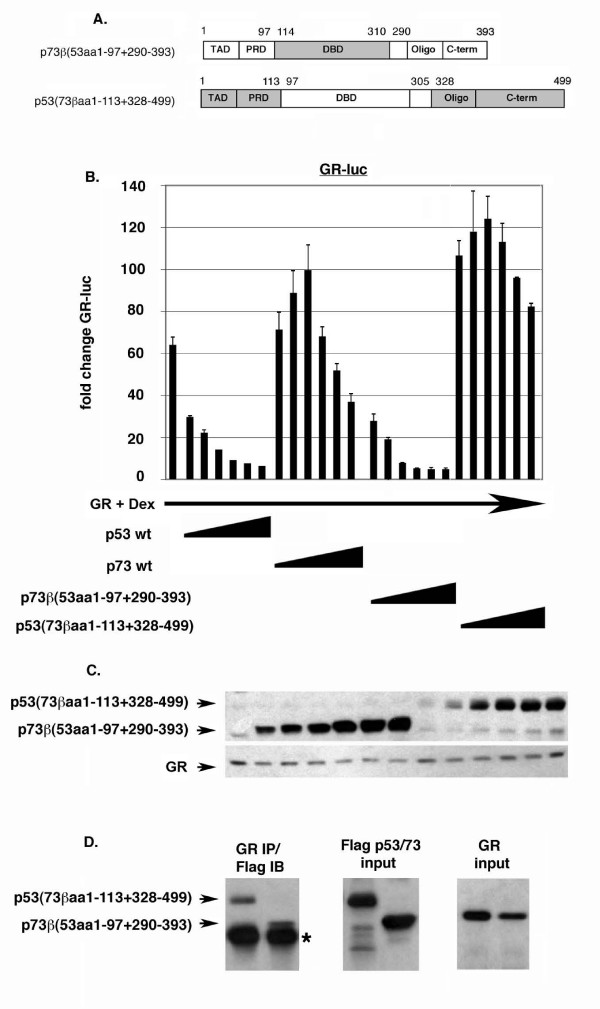
**N-terminal and C-terminal sequences in p53 allow inhibition of GR**. *A*, Schematic of p53 and p73 swap mutants in which N-terminal and C-terminal sequences have been swapped simultaneously. *B*, Saos-2 cells were transfected with the GR-responsive luciferase reporter GR-luc (200 ng), GR DNA (100 ng), and increasing amounts DNA encoding the indicated p53:p73 protein (50 – 1000 ng of each DNA). Transfected cells were treated with Dex (100 nM) as described earlier, and luciferase activity in transfected cell lysates was determined. Fold change in GR-luc activity is plotted (+/- s.e.m.) from multiple experiments compared to the activity of GR-luc alone, whose value is considered 1.0. *C*, Representative immunoblots show the level of each protein. *D*, (Left) Lysates from Saos-2 cells transiently expressing GR and the indicated p53/p73 swap mutants were immunoprecipitated with a GR antibody and examined by immunoblotting with an anti-Flag antibody (GR IP/Flag IB). Arrowheads indicate the position of the indicated p53/p73 swap mutant. The asterisk indicates the position of the antibody heavy chain used in the immunoprecipitation. (Right) Lysates were examined prior to immunoprecipitation to show the relative input amounts of each protein.

To explore the differences between p53 and p73 further, a second set of p53:p73 chimeric proteins were generated in which larger regions of the N- and C-terminus of each protein were swapped (Fig. 8A). Chimeric proteins were also generated in which large N-terminal and C-terminal regions of p53 and p73 were swapped simultaneously (clones 15 and 16, Fig. 8A). These chimeric clones displayed varying levels of transcriptional activity, as monitored by activation of the pG13-luc reporter (Fig. 8B). We first examined GR inhibition by the p53:p73 chimeric proteins in which both the oligo and C-terminal (C-term) sequences have been swapped (Fig. 9B). p53 with the p73 oligo and C-term [p53(73βaa328–499)] was still able to inhibit GR activity, though with a slightly lesser ability than p53 wt at low input amounts. p73 with the p53 oligo and C-terminal domain [p73β(53aa290–393)] could also inhibit GR. When equal protein expression levels were compared (as determined by densitometric scanning of films), [p73β(53aa290–393)] and [p53(73βaa328–499)] inhibited GR with comparable efficiencies. These results are consistent with those of Fig. 6 and indicate the different abilities of p53 and p73 to inhibit GR may be explained in part, but not fully, by differences in their C-terminal sequences, including the oligo and C-term domains. Next, we examined GR inhibition by the p53:p73 chimeric proteins in which both the N-terminal TAD and proline rich domain (PRD) have been swapped (Fig. 10B). p53 with the p73 TAD and PRD [p53(73βaa1–113)] was still able to inhibit GR activity, though less well than p53 wt or p53 with only the p73 TAD swapped (compare GR inhibition in Fig. 7C and Fig. 9B). In contrast, p73 with the p53 TAD and PRD [p73β(53aa1–97)] gained ability to inhibit GR. When equal protein expression levels were compared, [p73β(53aa1–97)] appeared to inhibit GR to a greater extent than [p53(73βaa1–113)]. These results indicate the different abilities of p53 and p73 to inhibit GR may be explained in part, but not fully, by differences in their N-terminal sequences, including the TAD and PRD domains. Finally, we examined GR inhibition by p53:p73 chimeric proteins in which both N-terminal and C-terminal sequences have been swapped simultaneously (Fig. 11B). Strikingly, p53 with both the p73 N-terminus and C-terminus [p53(73βaa1–113+328–499)] was completely unable to inhibit GR activity and, like p73 wt, activated GR slightly at lower input amounts. In contrast, p73 with both the p53 N-terminus and C-terminus [p73β(53aa1–97+290–393)] efficiently inhibited GR at all input amounts, and to an extent similar to p53 wt. It is important to note that both these clones displayed comparable levels of transcriptional activity (clones 15 and 16, Fig. 8B). Further, co-immunoprecipitation experiments demonstrated that both chimeric proteins could bind GR to equal extents (Fig. 11D), indicating their different abilities to inhibit GR did not result from differences in GR binding. We conclude that the different ability of p53 and p73 to inhibit GR results from differences in their N-terminal and C-terminal sequences, and is not due to differences in their transcriptional activity or GR-binding.

## Discussion

Binding between p53 and GR has been demonstrated *in vivo *and *in vitro*, and p53 and GR can inhibit each others transcriptional activity in transfected cells [27,28]. Moreover, activation of endogenous GR by Dex treatment inhibited p53 induced cell cycle arrest in stressed cells, and activation of endogenous p53 by DNA damage inhibited GR transcriptional activity [27,28]. These findings and others support the existence of negative cross-talk between the p53 and GR pathways. The GR-binding site in p53 has been mapped to the p53 DNA-binding domain (DBD) [28], and Dex treatement was reported to both enhance p53-GR binding and promote their proteasomal degradation to some extent. P53 and p73 are structurally similar and share high amino acid sequence identity in their DBD and other domains. However, to date, neither physical nor functional interactions between p73 and GR have been reported. In some but not all experiments, GR and p53 levels decreased upon Dex treatment in cells co-expressing both proteins, and this was partially blocked by proteasome inhibition. GR and p73 levels were also decreased in Dex-treated cells in some experiments (Fig. 1B). This suggests Dex treatment may promote proteasomal degradation of GR, p53, and p73. Our co-immunoprecipitation studies (Fig. 1C) indicate that p73 can also bind GR, similar to p53. Interestingly, binding between GR and p53 or p73β was most evident in cells treated with the proteasome inhibitor MG132. This suggests the ability to detect GR complexes with p53 or p73 may be limited by their proteasomal degradation. Alternatively, MG132 may stabilize one or more factors that enhance the interaction between GR and either p53 or p73.

GR can inhibit the transcriptional activity of p53. To test whether GR can also inhibit the transcriptional activity of p73, we monitored the effect of GR co-expression on p53 and p73-dependent activation of different luciferase reporter genes. GR inhibited transactivation of all three reporter genes by p53 or p73 to comparable extents. Thus, p53 and p73 can bind GR, and the transcriptional activity of both proteins is inhibited by GR. We also monitored the effect of p53 or p73 co-expression on the transcriptional activity of GR. In all experiments, p53 caused a pronounced inhibition of GR activity. Surprisingly, however, p73 was either unable to efficiently inhibit GR, or activated GR slightly (Fig. 3). Thus, p53 and p73 can bind GR, but only p53 and not p73 can efficiently inhibit GR activity. One interesting possibility based on these results is that the mechanism by which GR inhibits p53 and p73 may be different from the mechanism by which p53 or p73 inhibits GR. Binding to p53 or p73 may be sufficient for GR to inhibit p53 and p73 activity. However, binding alone may not be sufficient for p53 or p73 to inhibit GR, since p73 can bind GR just as p53 can, but is unable to efficiently inhibit GR activity.

Wasylyk and colleagues reported one way in which p53 can inhibit GR is through its activation of MDM2 [28]. According to this model, p53 activates MDM2 gene expression, and increased levels of MDM2 protein then promote the degradation of both p53 and GR. We observed that p53 could still efficiently inhibit GR activity either when endogenous MDM2 levels were decreased via siRNA, or in MDM2/p53 double-knockout cells. While MDM2 may contribute to GR inhibition by p53, our results indicate p53 can also inhibit GR through an MDM2-independent mechanism. P73 induced lower levels of MDM2 than did p53. However, p73 remained unable to inhibit GR activity when expressed with increasing amounts of MDM2, and MDM2 alone also had no effect on GR activity. These results indicate the different abilities of p53 and p73 to inhibit GR results from something other than differences in MDM2 levels.

We considered two possibilities might explain the different abilities of p53 and p73 to inhibit GR. First, p53 may contain one or more unique sequence elements that are absent from p73, and that allow p53 to inhibit GR. Second, p73 may contain one or more unique sequence elements that are missing from p53, and that prevent p73 from inhibiting GR. To investigate these possibilities, a series of p53:p73 chimeric proteins were generated in which corresponding regions of either protein have been swapped (Figs. 5A and 8A). The high degree of structural homology between p53 and p73 should allow one to switch the various domains of each protein without disrupting protein conformation. This approach was used previously by Yuan and colleagues to identify specific sequence elements in p53 that confer its degradation by MDM2 [34]. Our studies revealed that replacing C-terminal domains (oligo and C-term) in p53 with the corresponding domains of p73 had little effect, or only slightly diminished, the ability of p53 to inhibit GR. Conversely, deleting the unique C-terminus of p73, or replacing C-terminal domains (oligo and C-term) in p73 with the corresponding domains of p53 allowed p73 to inhibit GR to a large extent. Replacing the N-terminal domains (TAD and PRD) in p53 with the corresponding domains of p73 diminished the ability of p53 to inhibit GR, whereas replacing the N-terminal domains in p73 with the corresponding domains of p53 allowed p73 to inhibit GR to some extent. These results suggested that the different abilities of p53 and p73 to inhibit GR can be explained in part, but not fully, by individual differences in their N- and C-terminal sequences. Confirmation of this came from analysis of p53:p73 chimeric proteins in which their N- and C-terminal sequences were swapped simultaneously (Fig. 11). Replacement of p53s N- and C-terminal sequences with the N- and C-terminal sequences of p73 at the same time completely blocked its ability to inhibit GR, while replacement of p73s N- and C-terminal sequences with the N- and C-terminal sequences of p53 at the same time allowed it to efficiently inhibit GR. Importantly, the different ability of these chimeric proteins to inhibit GR was not due to differences in their transcriptional activity or ability to bind GR. The results suggest that p53 N- and C-terminal sequences contribute to its inhibition of GR, since these sequences cannot be replaced by the corresponding sequences of p73. In contrast, p73 N- and C-terminal sequences appear to prevent GR inhibition, since deletion of C-terminal sequences from p73 restored some ability to inhibit GR, and replacing p53s N- and C-terminal sequences with those of p73 blocked p53 from inhibiting GR. This raises the question of how the N- and C-terminal sequences of p53 or p73 might participate in GR inhibition. A recent study suggested that p53 could repress transcription of the IGFBP-1 gene through recruitment of a histone deacetylase (HDACs) [38]. Interestingly, this recruitment of HDAC required both N-terminal and C-terminal sequences in p53. Perhaps p53 can bind GR on promoter DNA and repress its activity through N- and C-terminal sequences and HDAC recruitment. p73 might lack the ability to recruit HDAC protein(s) or only recruit HDACs inefficiently, accounting for its inability to inhibit GR.

## Conclusion

p53 and p73 transcriptional activity is inhibited by glucocorticoid receptor (GR) co-expression. However, whereas wild-type p53 efficiently inhibits GR transcriptional activity, p73 is either unable to efficiently inhibit GR, or increases GR activity slightly. These differences are not due to differences in the ability of p53 and p73 to either bind GR, or activate MDM2 gene expression. Analysis of multiple p53:p73 chimeric proteins revealed that the differential ability of p53 and p73 to inhibit GR is due to differences in their N-terminal and C-terminal sequences.

## Methods

### Plasmid DNAs

HA-tagged and Flag-tagged p53 wt DNAs were described previously [39]. HA-tagged human p73β DNA was from Frank Mckeon (Harvard Medical School). Flag-tagged p73β was generated by PCR amplifying p73β sequences from HA p73β DNA and cloning them into Bam HI and Xba I sites downstream of the Flag epitope. p53(73βaa1–54), and p73β(53aa1–45) DNAs were gifts from Zhimin Yuan (Harvard School of Public Health. All other DNAs encoding human p53:p73β chimeric proteins were generated by two-step PCR method, as described previously [34]. All chimeric DNAs were confirmed by DNA sequencing. Oligonucleotide sequences used in cloning are available on request. DNA encoding glucocorticoid receptor (GR) and GR-responsive luciferase reporter gene (GR-luc) was from E.A. Thompson (University of Texas Medical Branch, Galveston, TX). GR-luc contains two copies of a GR-responsive element from the rat angiotensin gene upstream of minimal promoter. PG13-luc DNA was from Bert Vogelstein (Johns Hopkins). P21-luc and MDM2-luc DNA were from Moshe Oren (Israel). DNA encoding siRNA against MDM2 was described [40] and was from Ruiwen Zhang (University of Alabama-Birmingham).

### Cell culture and transfections

Saos-2 cells are a p53-null human osteosarcoma cell line. MDM2/p53 double knockout (DKO) mouse embryo fibroblasts were from Rudy Alarcon (Stanford University). Cell lines were maintained at 37°C in Dulbeccos Modified Eagles Medium (DMEM) supplemented with 10% fetal bovine serum and antibiotics (1% penicillin and streptomycin). Transfections in Saos-2 or MDM2/p53 DKO cells were done using Fugene-6 transfection reagent (Roche) according to the manufacturers protocol when cells were approximately 60% confluent. Total DNA in each transfection was equalized by addition of empty plasmid. Dexamethasone (Dex) was purchased from Sigma and was dissolved in ethanol at a concentration of 1 mM. Dex was added to cells at a final concentration of 100 nM to activate GR, and cells harvested 20–24 hrs later. Where indicated, the proteasome inhibitor MG132 (Boston Biochem) was added to a final concentration of 30 μM and the cells incubated for an additional 6 hrs before harvesting.

### Immunoprecipitations and Immunoblotting

To harvest cell lysates for immunoblotting, cells were rinsed with 2 ml PBS and then scraped into 500 μl lysis buffer (50 mM Tris pH 7.5, 150 mM NaCl, 0.5% NP40, PMSF, leupeptin) and transferred to microfuge tubes. The cells were then incubated on ice for 30 min with occasional vortexing, and spun at 4°C, 14,000 rpm for 15 min to remove cellular debris. For co-immunoprecipitations, lysates were immunoprecipitated overnight with 200 ng anti-GR polyclonal antibody (clone E-20, Santa Cruz Biotechnology). GR immunoprecipitates were isolated on protein A agarose beads as described previously [39]. For immunoblotting, protein lysates or GR immunoprecipitates were resolved by sodium dodecyl sulfate-polyacrylamide gel electrophoresis (SDS-PAGE) and transferred to PVDF membranes for immunoblotting. Antibodies used in immunoblotting included anti-HA monoclonal antibody (HA.11, Covance), anti-Flag monoclonal antibody Ab-5 (Sigma-Aldrich), anti-GR polyclonal antibodies (E-20 and P-20, Santa Cruz Biotechnology), anti-MDM2 monoclonal antibody SMP-14 (Santa Cruz Biotechnology).

### Luciferase Assays

To monitor p53 and p73 transcriptional activity, Saos-2 cells were transfected with 100 ng of each luciferase reporter DNA (pG13-luc, p21-luc, MDM2-luc) and 50 ng DNA encoding Flag p53 wt or Flag p73β wt. In some cases, GR DNA was included in the transfection (100, 250, 500, or 1000 ng) and cells were treated with Dex (100 nM) for 16–20 hrs before harvesting. To monitor GR transcriptional activity, cells were transfected overnight with 200 ng GR-luc DNA and 100 ng DNA encoding GR. Cells were then treated with Dex (100 nM) for 20–24 hrs before harvesting. In some cases, p53 wt or p73 wt, or the various chimeric proteins were included in the transfection. PRL-TK DNA (5 ng) was included in each transfection as a normalization control. Luciferase activity was monitored using the dual-luciferase reporter assay kit (Promega Corp.), according to the manufacturers protocol. P53 and p73 protein expression levels were compared in these luciferase experiments by densitometric scanning of films using Image-J software. This allowed comparison of GR-luc activity under conditions of comparable p53 and p73 protein expression.

## Competing interests

The author(s) declare that they have no competing interests.

## Authors' contributions

LZ carried out the bulk of the experiments described in this study. LN generated the p53:p73 chimeric proteins and assisted in data analysis and experimental design. CGM directed the laboratory work, interpreted the data, and wrote the manuscript.
